# Atmospheric transformation of plant volatiles disrupts host plant finding

**DOI:** 10.1038/srep33851

**Published:** 2016-09-21

**Authors:** Tao Li, James D. Blande, Jarmo K. Holopainen

**Affiliations:** 1 Department of Environmental and Biological Sciences, University of Eastern Finland, Kuopio Campus, PO Box 1627, FI-70211, Kuopio, Finland

## Abstract

Plant-emitted volatile organic compounds (VOCs) play important roles in plant-insect interactions. Atmospheric pollutants such as ozone (O_3_) can react with VOCs and affect the dynamics and fidelity of these interactions. However, the effects of atmospheric degradation of plant VOCs on plant-insect interactions remains understudied. We used a system comprising *Brassica oleracea* subsp. *capitata* (cabbage) and the specialist herbivore *Plutella xylostella* to test whether O_3_-triggered VOC degradation disturbs larval host orientation, and to investigate the underlying mechanisms. Larvae oriented towards both constitutive and larva-induced cabbage VOC blends, the latter being the more attractive. Such behaviour was, however, dramatically reduced in O_3_-polluted environments. Mechanistically, O_3_ rapidly degraded VOCs with the magnitude of degradation increasing with O_3_ levels. Furthermore, we used Teflon filters to collect VOCs and their reaction products, which were used as odour sources in behavioural tests. Larvae avoided filters exposed to O_3_-transformed VOCs and spent less time searching on them compared to filters exposed to original VOCs, which suggests that some degradation products may have repellent properties. Our study clearly demonstrates that oxidizing pollutants in the atmosphere can interfere with insect host location, and highlights the need to address their broader impacts when evaluating the ecological significance of VOC-mediated interactions.

Volatile organic compounds (VOCs) emitted by plants are known to serve as important infochemicals in multitrophic interactions^1^. Herbivorous insects, for example, exploit plant VOCs to identify different plant species in order to differentiate host plants from non-hosts, and to evaluate the suitability of different available hosts^2^. However, the effectiveness of VOC-mediated plant-insect interactions strongly depends on the surrounding environment and is being progressively reduced with increasing intensity of air pollution.

Most plant VOCs, especially monoterpenes and sesquiterpenes, have relatively short atmospheric lifetimes because they react readily with atmospheric oxidant pollutants such as ozone (O_3_), hydroxyl radicals (OH) and nitrate radicals (NO_3_)^3^,^4^. The O_3_-reactive semivolatile diterpene *cis*-abienol, exuded by the glandular trichomes of tobacco plants, significantly reduced O_3_ flux through open stomata and into a tobacco leaf, while ozonolysis reaction products of *cis*-abienol, formaldehyde, methyl vinyl ketone (MVK) and 4-oxopentanal, were observed to increase in the leaf boundary gas phase^5^. Therefore, the informative value of cues emitted by plants for surrounding organisms may be altered in an oxidant-polluted environment. Despite the numerous studies on plant VOCs as efficient mediators of interactions between plants and their surroundings, the implications of air pollution for these chemically mediated interactions are often ignored and have only recently begun to be more appreciated^6^,^7^,^8^,^9^. Both theoretical and empirical studies have indicated that pollinator foraging distance can be dramatically reduced with increasing concentrations of atmospheric oxidants^3^,^10^,^11^,^12^. O_3_ has also been shown to affect the efficiency of a parasitoid in finding its prey^13^,^14^, and of a florivore in finding its host flowers^15^. In addition, our previous studies have found that increasing O_3_ levels significantly reduce the distance over which plant-plant communication occurs^16^,^17^. All these studies have ascribed the reduced effectiveness of VOC-mediated multitrophic interactions to the degradation of putative bioactive VOCs and the disintegrity of active VOC blends. Taken together, these studies suggest that increasing levels of O_3_ and other air pollutants could have dramatic effects on volatile-mediated infochemical networks and highlight the need to address the broader impacts of pollutants in studies of chemical ecology and take a more integrative approach to thinking on the impacts of pollutants.

Not only can atmospheric oxidants degrade bioactive VOCs, but photochemical oxidation of plant VOCs also leads to formation of a wide range of gaseous reaction products and secondary organic aerosols (SOA)^5^,^18^,^19^,^20^. These oxidation products and/or aerosol particles may have ecological consequences for plant-insect interactions once they are rained out or directly deposit to plant surfaces via dry or wet deposition. For example, some studies have shown that plants are capable of taking up and metabolizing oxygenated VOCs formed during the photochemical oxidation of certain monoterpenes^18^,^19^,^21^. In these studies, several genes related to plant defence against oxidative stress were found to be up-regulated when plants were exposed to methyl vinyl ketone and macroclein^18^,^22^, which accounts for more than 80% of reaction products of isoprene in the first phase of reaction in the atmosphere. Interestingly, these genes have also been shown to be implicated in plant defence against herbivory^22^. This hints that deposition of condensable gaseous and aerosol reaction products of plant VOCs may impact on plant growth and defence, which in turn may affect plant-insect interactions. Yet, no efforts have been directed towards understanding the potential effects of atmospheric reaction products on plant-insect interactions.

In the present study, we used cabbage (*Brassica oleracea*) and the diamondback moth, *Plutella xylostella*, as a model system to determine whether *P. xylostella* larvae utilize plant volatiles as olfactory cues in search of host plants and to evaluate whether and how atmospheric oxidizing pollutants impact such process by degrading volatile cues and forming reaction products. *P. xylostella* is an insect herbivore specialized on *Brassica* plants and is considered the most destructive insect pests of cruciferous crops throughout the world^23^. It is known to utilize plant chemistry in the process of host selection and acceptance. For instance, oviposition and feeding stimulation by glucosinolates, the characteristic chemical constituents of *Brassica* plants, and their volatile hydrolysis products like isothiocyanates, have been documented in *P. xylostella*^23^,^24^. Furthermore, several studies have shown that *P. xylostella* adults may also exploit other host plant VOCs such as terpenoids and green leaf volatiles (GLVs) when searching for an oviposition site^25^,^26^. However, no attempts have been made to characterize the orientation responses of *P. xylostella* larvae to host plant VOCs. Newly hatched *P. xylostella* larvae have been reported to move from oviposition sites to feeding sites^27^ and therefore may require a suitable olfactory cue to locate the host plants or plant parts of high quality, as is the case with larvae from many other Lepidopteran and Coleopteran species^28^,^29^,^30^,^31^,^32^.

More importantly, we investigated whether increasing levels of tropospheric O_3_ can interfere with host orientation by degrading volatile/semivolatile cues. Ground-level O_3_ is recognised as the most important rural air pollutant due to drift from more urbanised areas^33^. At present, peak O_3_ concentrations in the vicinity of urbanized areas commonly exceed 100 ppb and occasionally reach 200 ppb^33^,^34^. Furthermore, we attempted to separate the effects of O_3_-initiated loss of putative bioactive volatiles from the effects of O_3_-initiated reaction products, particularly SOA particles.

## Results

### Role of plant volatiles in host-orientation behaviour of *P. xylostella* larvae

**Figure 1:**
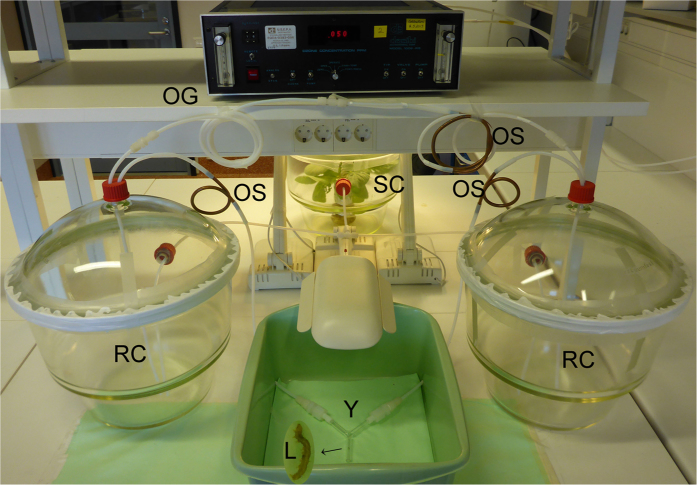
Experimental setup for the demonstration of chemical reaction between plant VOCs and O_3_, and dual-choice olfactory bioassay. SC: odour source chamber; RC: reaction chamber; OG: ozone generator; OS: ozone scrubber (KI coated copper tube); Y: Y-tube olfactometer; L: *P. xylostella* larva.

**Figure 2:**
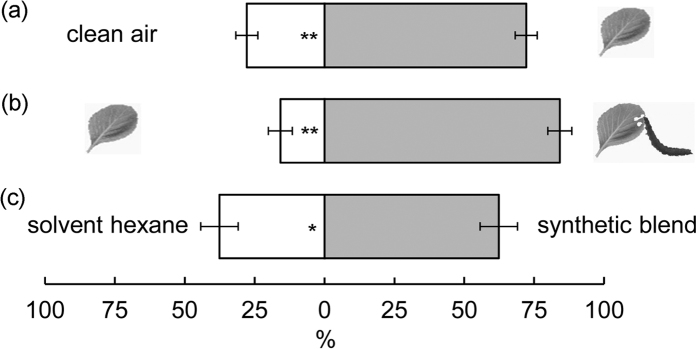
Orientation (percentage ± se) of *P. xylostella* larvae to VOCs from (**a**) healthy cabbage plants vs clean air, (**b**) larva-infested vs healthy cabbage plants, and (**c**) a synthetic bouquet resembling the larva-induced blend vs a hexane control in Y-tube olfactometers. The number of responding larvae/the total number of larvae tested is 110/128 (**a**), 123/134 (**b**), and 65/132 (**c**). Asterisks indicate a preference which is significantly different from an equal distribution within a choice test: *P ≤ 0.05; **P ≤ 0.001.

To test whether *P. xylostella* larvae utilize plant VOCs as host location cues, we determined the VOC emissions of cabbage plants with and without feeding damage and observed olfactory responses of larvae to the emitted VOC blends. Cabbage seedlings constitutively released several terpenoids and one sulphur-containing compound. In the Y-tube olfactometer bioassays (Fig. 1), the number of *P. xylostella* larvae orienting towards VOCs from undamaged plants was significantly higher than the number of larvae orienting towards charcoal filtered air (Fig. 2a; *P* < 0.001). When offered a choice between VOCs emanating from undamaged and *P*. *xylostella*-infested plants, larvae showed a strong preference for the latter (Fig. 2b; *P* < 0.001). Both PCA and independent t-tests revealed that the VOC blends emitted by infested plants differed significantly from those emitted by undamaged plants both qualitatively and quantitatively (see Supplementary Figs S1–S3). Specifically, infested plants not only increased emissions of constitutively emitted compounds, but also a de novo synthesized and emitted homoterpene (*E*)-DMNT and two GLVs (*Z*)-3-hexenol and (*Z*)-3-hexenyl acetate, which contributed most to the differences between control and infested plants. To examine the importance of GLVs and terpenoids in host recognition, we tested the olfactory responses of larvae to a synthetic blend comprising two GLVs and seven terpenoids that mimics the ratios observed in the volatile blends emitted by infested plants. In this case, larvae oriented more frequently toward the synthetic blend than clean air (Fig. 2c).

### Impact of O_3_-transformed plant volatiles on host orientation of *P. xylostella* larvae

**Figure 3:**
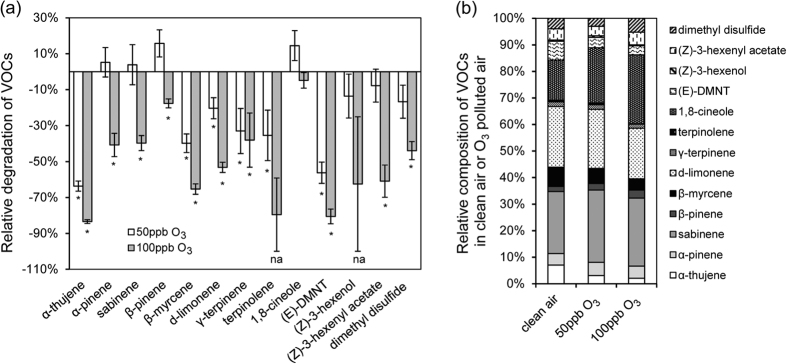
Degradation of *P. xylostella*-induced cabbage VOCs by O_3_: (**a**) relative reduction (mean ± se) in VOC concentrations and (**b**) changes in relative blend compositions after exposure to different O_3_ concentrations. Asterisks indicate a significant reduction at each O_3_ level (na: no statistical analysis because of zero-inflated data distribution). (*E*)-DMNT: (*E*)-4,8-dimethyl-1,3,7-nonatriene. *P ≤ 0.05.

**Figure 4:**
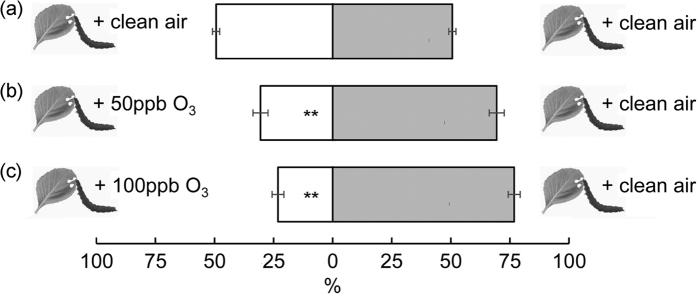
Attraction (percentage ± se) of *P. xylostella* larvae to *P. xylostella*-induced cabbage VOC blends mixed with O_3_ (50 or 100 ppb) or clean air in Y-tube olfactometers. The number of responding larvae/the total number of tested larvae in (**a**–**c**) is 85/98, 109/133 and 104/117, respectively. Asterisks indicate a preference which is significantly different from an equal distribution within a choice test: **P ≤ 0.001.

To test whether O_3_ degrades host volatile cues and consequently interferes with host orientation behaviour, we mixed *P*. *xylostella*-induced VOC blends with O_3_, followed by olfactory bioassays and VOC analyses (Fig. 1). O_3_ reacted with many volatile compounds, breaking down most of the compounds and rendering the O_3_-oxidized blends markedly different from the original ones (Fig. 3 and see Supplementary Figs S1, S4–S6). At 100 ppb O_3_, for instance, ozonolysis increased the relative proportion of the low reactivity compounds 1,8-cineole and β-pinene while decreasing the relative proportion of the moderately high reactivity compounds α-thujene, β-myrcene and (*E*)-DMNT, which was mainly responsible for the separation between the O_3_-oxidized and original iVOC blends (Fig. 3 and see Supplementary Fig. S6). The extent of VOC degradation appeared to increase with increasing O_3_ levels. In the Y-tube bioassays, larvae preferred volatiles exposed to filtered air over those mixed with O_3_, which was observed at both O_3_ levels tested (Fig. 4; *P* < 0.001). When O_3_ supply to the reaction chamber was eliminated with O_3_ scrubbers, larvae had no preference for either side (Fig. 4a; *P* = 1.00).

### Effects of O_3_-initiated reaction products on orientation responses of *P. xylostella* larvae

**Figure 5:**
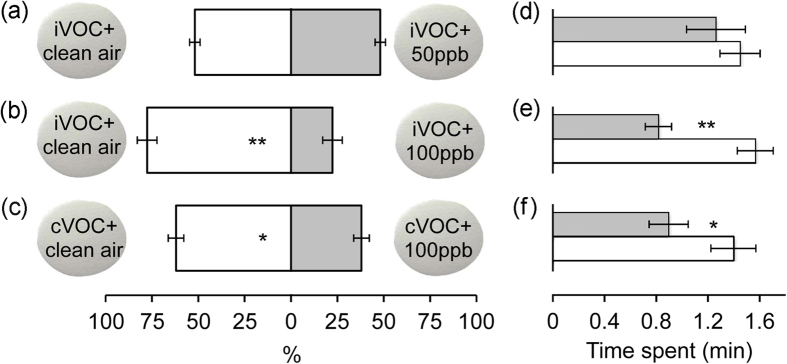
Orientation of *P. xylostella* larvae in Petri dishes towards Teflon filters that had previously been exposed to cabbage VOCs (iVOC: *P. xylostella*-induced VOC; cVOC: constitutive VOC) mixed with O_3_ (grey bars; 50 or 100 ppb) or clean air (white bars). (**a**–**c**) Percentage (mean ± se) of larvae choosing filters. The number of responding larvae/the total number of tested larvae in (**a**–**c**) is 80/120, 73/121 and 77/121, respectively. Asterisks indicate a significant preference based on two-sided binomial tests. (**d**–**f**) Mean time spent on filters (±se). Data in (**d**–**f**) correspond to observations in (**a**–**c**), respectively; the number of larvae observed (grey|white) is 22|26, 12|32 and 23|31, respectively. Asterisks denote a significant difference according to Mann-Whitney *U* tests. *P ≤ 0.05; **P ≤ 0.001.

In an attempt to differentiate the effects of reaction products and O_3_-triggered disintegration of biologically active VOC blends, we collected reaction products, mainly SOA, onto Teflon filters, followed by behavioural tests. The number of larvae climbing onto Teflon filters that had been exposed to volatiles mixed with O_3_ was significantly lower than those climbing onto Teflon filters that had been exposed to volatiles that were not mixed with O_3_ (Fig. 5 and see Supplementary Fig. S7). This response was found for both induced (Fig. 5b; *P* < 0.001) and constitutive (Fig. 5c; *P* = 0.03) VOC blends. In addition, larvae spent much less time searching on filters exposed to volatiles mixed with O_3_ than those not mixed with O_3_ (Fig. 5e,f; *P* < 0.001 and *P* = 0.039 for induced and constitutive VOC blends, respectively). However, this effect was only observed at 100 ppb and not at 50 ppb (Fig. 5a,d), although under both O_3_ scenarios several terpenoids were degraded to varying extents.

**Figure 6:**
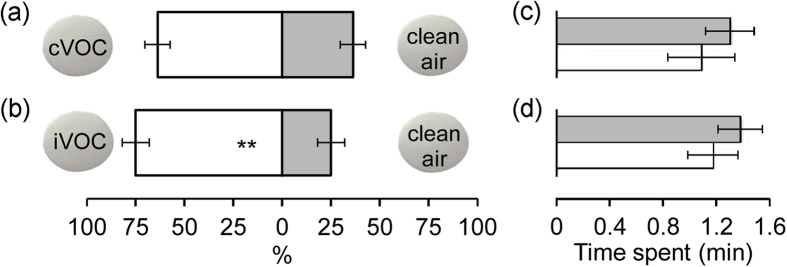
Orientation of *P. xylostella* larvae in Petri dishes towards Teflon filters that had been exposed to cabbage VOCs (while bars; iVOC: *P. xylostella*-induced VOC; cVOC: constitutive VOC) or clean air (grey bars). (**a**,**b**) Percentage (mean ± se) of larvae choosing filters. The number of responding larvae/the total number of tested larvae in (**a**,**b**) is 64/160 and 65/112, respectively. Asterisks indicate a significant preference based on two-sided binomial tests: **P ≤ 0.001. (**c**,**d**) Mean time spent on filters (±se). Data in (**c**; grey|white = 12|16) and (**d**; grey|white = 17|47) were derived from a subset of observations in (**a**,**b**), respectively.

To further test if volatiles can condense onto the filter surface and then affect behavioural responses after re-release, we compared the preferences of larvae for filters exposed to plant volatiles versus clean air. Larvae preferred filters exposed to induced VOC blends to those exposed to clean air (*P* < 0.001), but there was no significant preference for filters exposed to constitutive VOC blends over those exposed to clean air (*P* = 0.169) (Fig. 6a,b). In both cases, the amount of time spent walking on filters was very similar (Fig. 6c,d; *P* = 0.422 and *P* = 0.412 for induced and constitutive VOC blends, respectively). Additionally, comparisons across different filter disc bioassays revealed that larvae devoted the least amount of time to foraging on filters exposed to O_3_-transformed VOC blends, followed by filters exposed to clean air and then original VOC blends (see Supplementary Fig. S8).

## Discussion

The primary aim of this study was to investigate how O_3_ pollution may affect volatile-mediated host location by arthropod herbivores. Our results reveal that higher O_3_ concentrations, in excess of 50 ppb, break down several plant VOCs, alter the ratio of components in the blend, and eventually reduce the ability of herbivores to orient towards the VOC blend emitted from their host plants. Most intriguingly, we showed that certain reaction products formed in the process of VOC degradation may have repellent effects and may hence have contributed to the reduction in host searching efficiency. Our findings provide new information concerning the impacts of air pollution on an ecologically important VOC-mediated process and shed new insight into the underlying mechanisms.

Many studies have found that *P. xylostella* adults make use of plant VOCs when searching for host plants on which to lay their eggs^24^,^25^,^26^,^27^,^35^. Our study extends these findings by illustrating for the first time that *P. xylostella* larvae may also utilize volatile cues to recognize the most suitable food sources. Similarly, an increasing number of studies on lepidopteran and coleopteran species have demonstrated that larvae are capable of orienting towards their host plants by using plant odours as olfactory cues^29^,^30^,^31^,^36^, or avoid their host plant if contaminated with neighbouring plant volatiles^37^, though larvae of many insect species are more sedentary and have simpler olfactory systems than their adult counterparts^38^.

In the Brassicaceae, non-volatile glucosinolates and their volatile hydrolytic breakdown products such as isothiocyanates have been documented to stimulate *P. xylostella* adult oviposition and larval feeding activity^23^. However, other volatile compounds may also be implicated in *P. xylostella* host orientation. Pivnick *et al*.^25^ found that solvent fractions of *B. juncea* leaves were attractive to *P. xylostella* females whether or not isothiocynanates were present, with a terpenoid-containing fraction being most attractive. Reddy and Guerrero^26^ showed that GLVs from *B. oleracea* subsp. *capitata*, in particular (*Z*)-3-hexenol and (*Z*)-3-hexenyl acetate, acted either alone or synergistically with sex pheromone to attract both *P. xylostella* females and males. In our study, we could not detect any breakdown products of glucosinolates, but we found that a synthetic blend reconstituting the major terpenoids and GLVs emitted by damaged plants was attractive to *P. xylostella* larvae. Collectively, these studies indicate the importance of terpenoids and GLVs in the *P. xylostella* host-location process.

The effects of O_3_ pollution on the integrity of biologically relevant VOC blends and the effects on insect olfactory behaviour were striking. Consistent with earlier studies^3^,^11^,^13^,^14^,^17^, we found that O_3_ rapidly degraded cabbage VOCs, with the magnitude of degradation increasing with O_3_ level. This led to drastic changes in both the concentrations and the ratios of different compounds in the blend. At 100 ppb O_3_, for instance, the concentrations of sabinene and d-limonene, the two most dominant cabbage VOCs, were reduced by approximately 40% and 53%, respectively.

Foraging *P. xylostella* larvae oriented toward VOC blends not mixed with O_3_ as opposed to those mixed with O_3_. Such olfactory responses were more apparent at an O_3_ concentration of 100 ppb than at 50 ppb, which matched with the higher degradation rate observed at 100 ppb relative to 50 ppb. Similarly, our recent investigation of *P. xylostella* adults^17^ showed that adsorption of broccoli VOCs onto neighbouring plants stimulated oviposition, mediating associational susceptibility, but this process was eliminated at an O_3_ level of 80 ppb. Likewise, an earlier study found that O_3_ levels above 80 ppb rendered the adults of the striped cucumber beetle (*Acalymma vittatum*) unable to distinguish between ozonated and non-ozonated VOC blends emitted by its host plant *Cucurbita foetidissima*^15^. In all cases, O_3_-triggered loss of the biological activity of VOC blends is most likely caused by reduced concentrations and/or altered ratios of different components in the VOC blends. Previous studies have shown that changes in the composition of volatile blends and/or shifts in the ratios of constituents, for example by spiking host plant VOC blends with synthetic compounds, can result in a disruption of the orientation of herbivorous insects to the host plants^39^. Taken together, these studies indicate that VOC-mediated plant-herbivore interactions could be significantly compromised in O_3_-polluted environments, as O_3_ can destruct the integrity and strength of bioactive VOC blends to such an extent that they no longer stimulate the insect olfactory system.

While O_3_-initiated breakdown of plant VOCs may seemingly have a beneficial side effect on plants through reduced uptake of phytotoxic ozone^5^ and decreased chemical apparency to herbivores as shown here, elevated tropospheric O_3_ levels can dramatically compromise many other VOC-mediated ecological interactions^9^, likely leading to an overall net negative impact on plant fitness. For example, both the flower-searching behaviour of pollinators and the host/prey-searching behaviours of carnivorous arthropods have been shown to be reduced in efficiency by ozone^3^,^6^,^11^,^13^,^14^. Elevated O_3_ concentrations have also been shown to impede volatile-mediated plant-plant communication^16^,^17^. Moreover, in nature, the presence of O_3_ can also alter plant VOC emission patterns and influence the ability of insects to perceive and respond to VOC cues^9^.

Apart from O_3_-initiated degradation of bioactive volatile compounds, the formation of new products in this process may also play an important but overlooked role in plant-insect interactions. This notion is tentatively supported by our finding that at an O_3_ level of 100 ppb, *P. xylostella* larvae oriented significantly less frequently toward the Teflon filters exposed to O_3_-transformed VOC blends and spent much less time searching on them compared to the filters exposed to original VOC blends. This holds true irrespective of whether herbivore-induced or constitutively emitted VOC blends were tested. It is possible that certain compounds in the original VOC blends might have adsorbed onto the filters and contributed to their attractiveness, since filters exposed to herbivore-induced VOC blends were found to be preferred over clean air-exposed ones. However, when filters were exposed to constitutive VOC blends, larvae did not show any preference for either VOC- or clean air-exposed filters, and in both cases larvae spent similar amounts of time searching on these filters. Moreover, comparing data across different filter bioassays indicates that search times were shorter for filters exposed to O_3_-transformed VOCs than those exposed to clean air. Together, these observations hint at the presence of certain repellent degradation products or unattractive ratios of host plant volatiles on the filters exposed to O_3_-transformed VOC blends, and the avoidance of these compounds by *P. xylostella* larvae.

While we attempted to analyze the compounds deposited on the Teflon filters, we did not succeed due to technical limitations. Consequently, we are unable to directly relate the observed insect behavioural responses to the formation of reaction products. A previous study that used the same cabbage cultivar as in the present study, confirmed the formation of SOA nanoparticles during ozonolysis of inducible plant VOCs^40^. Nonetheless, our study suggests that some low volatility oxidation products of cabbage VOCs may partition into the gas phase to interfere with the short-range olfactory response of *P. xylostella* larvae, and might also disturb the larval gustatory response when condensed onto the plant surfaces. Future studies that attempt to characterize reaction products and assess insect olfactory and gustatory responses and feeding performance using synthetic compounds would provide new insights into the identity and functioning of oxidation productions of plant VOCs.

The reaction products formed during atmospheric degradation of plant VOCs can be extraordinarily diverse and can readily participate in the formation of SOAs by condensation between themselves or through interaction with seed particles. For example, ozonolysis of limonene, the major terpenoid found in our study, has previously been shown to yield nearly 1200 different organic compounds and form a considerable amount of aerosol particles^41^. Semi- and low-volatile oxidation products, in particular when they come in the form of aerosol particles, can be deposited on plant surfaces; those with high volatility can desorb back to the gas phase^5^. These reaction products may contain repellent or even toxic compounds that directly affect host acceptance by insects, or volatiles that mask the target VOC odour that can be interpreted by a potential insect receiver, but in some cases may also enhance insect olfactory and gustatory responses. However, we are unware of studies that have directly tested these hypotheses, though a great effort has recently been made to learn more about the fates of biogenic VOCs and the formation of SOAs in the atmosphere^19^,^20^,^42^. Thus far, there are only a few lines of indirect evidence for the potential biological effects of VOC degradation products, which stem from observations on a few normal constituents of the environment that are produced endogenously in plants, animals and humans. For instance, formaldehyde, a common degradation product of VOCs^5^, has been shown to reduce activity of herbivorous slugs when encapsulated in fertilizer pellets and released from soil^43^, but stimulate growth of moth larvae when added to artificial diet^44^. Methyl vinyl ketone, another common ozonolysis product of volatile terpenoids^5^,^45^, is cytotoxic and can induce plasma membrane damage and decreased viability of animal cells^46^. Acetone, one of the primary carbonyls formed from O_3_ reaction^47^, has been reported to be relatively toxic to some herbivorous insects^48^.

In summary, our study clearly demonstrates that *Plutella xylostella* larvae can orient towards their host plants through olfaction, and that damaged plants are more attractive. However, such olfactory-guided orientation behaviour can be disrupted dramatically in O_3_-polluted environments, with the likely mechanisms being the altered composition and ratio of key components in the VOC blend due to O_3_-triggered degradation of VOCs, as well as the formation of some reaction products with possible repellent properties. It has to be noted that results from our study featuring a single atmospheric oxidant may not accurately describe the real world, where VOCs are susceptible to oxidation by multiple atmospheric oxidants including OH and NOx radicals in addition to O_3_, although ozonolysis often leads also to OH radical formation^42^. Simultaneous reaction with these oxidants may potentially lead to enhanced levels of reaction products and then enhanced biological effects that are not accounted for by studies using single oxidants alone. Future research should be directed toward addressing the role of these atmospherically relevant oxidizing systems on VOC degradation and SOA formation, as well as concomitant ecological consequences.

## Methods

### Plant and insect materials

Cabbage (*Brassica oleracea* subsp. *capitata* cv Lennox) and broccoli (*Brassica oleracea var.*) seeds were sown in a peat/sand mixture (3:1, v/v) in 1-L square pots and maintained under natural light in a greenhouse. Plants were grown for 4–6 weeks and typically had 4–5 expanded leaves, before being used in experiments. The majority of this study was performed using cabbage, unless specified otherwise.

Diamondback moth *Plutella xylostella* was reared on broccoli in cages made from acrylic and polyester gauze (60 × 33 × 33 cm, external dimensions) at 25 °C, 50% relative humidity, and 16/8 h light/dark photoperiod. Second to third instar larvae were used in all behavioural tests and were starved for 2–6 h before they were used in trials.

### Experimental manipulation

#### Experiment 1: Role of plant volatiles in host-orientation behaviour of P. xylostella larvae

To assess whether *P. xylostella* larvae can use plant volatiles as host-location cues, we carried out Y-tube olfactometer bioassays to compare their olfactory responses to VOCs from healthy plants versus clean air or from *P. xylostella*-infested plants versus healthy plants. We used detached plants instead of potted plants because this procedure better allows confounding volatiles from the rhizosphere and the potting substrate to be excluded. Plants were cut above the soil line and stems of individual plants were inserted into 15-ml glass bottles containing water. Infested plants were prepared by placing 25 mixed-instar *P. xylostella* larvae on each plant and allowing them to feed for 24 h. To avoid any effects of larva-derived odours, larvae and faeces were removed from leaves before the start of Y-tube bioassays (see below). Experiments were repeated over several days with 28–32 larvae tested per plant pair per day, and in total, four plant pairs were assessed per odour source combination. During these experiments, headspace volatile analysis from each odour source was conducted.

A synthetic volatile blend that mimics the release rates and ratios of the major terpenoids and GLVs emitted by the detached *P. xylostella*-induced cabbage plants was also tested. The synthetic blend was prepared in hexane and consisted of 25.0 ng d-limonene, 19.6 ng sabinene, 11.0 ng 1,8-cineole, 8.0 ng β-myrcene, 5.6 ng (*E*)-DMNT, 3.0 ng α-pinene, 1.4 ng β-pinene, 10.6 ng (*Z*)-3-hexenyl acetate and 1.0 ng (*Z*)-3-hexenol per μl hexane. Purity of all compounds was >98%. We applied 2 μl of the synthetic blend or 2 μl solvent to small rolls of filter paper. The paper rolls were allowed to dry for 1 min and then placed in 1-L glass jars for use as odour sources in olfactory tests. Each pair of paper rolls was assessed for only 1 h, and experiments were repeated eight times, each with newly-prepared paper rolls.

Y-tube bioassays were conducted using small glass Y-tube olfactometers (60 mm stem length, 60 arm length, 10 mm inner diameter), as described previously by Carroll *et al*.^29^. Compressed air, purified by an activated charcoal filter and MnO_2_ O_3_ scrubber, was split evenly into two airstreams with a Teflon T junction. For experiments with the synthetic blend, humidified air was used. Each airstream passed into a 1-L airtight glass vessel containing an odour source and then flowed into an arm of the olfactometer at a flow rate of 250 ml min^−1^. Teflon tubing was used to connect the different parts of the system. The olfactometer was placed inside a green plastic box which was illuminated from above and covered with aluminium foil during the bioassays to exclude visual cues and reduce visual bias. A single larva was released at the downwind base of the Y-tube, and its choice of an odour was scored when it reached the end of either source arm and did not return within at least 30 s. Larvae that did not satisfy these requisites within 5 min were judged as non-responsive and excluded from the statistical analysis. To account for any potential effect of associative learning and the traces left by the larvae on the choices made, each larva was tested only once and clean Y-tubes were used for each test. To compensate for any unforeseen asymmetry inherent to the experimental setup, we alternated odour sources after every 7–10 (plant odours) or 5 (synthetic blend) trials. During experiments, all glass vessels were thoroughly cleaned with ethanol and water, and oven-dried at 120 °C for a minimum 30 mins.

#### Experiment 2: Impact of O_3_-transformed plant volatiles on host orientation of P. xylostella larvae

O_3_ is known to react rapidly with many VOCs, disturbing volatile-mediated ecological interactions. To determine whether O_3_ can also interfere with host orientation of *P. xylostella* larvae through reacting with host volatiles, we observed the olfactory responses of *P. xylostella* larvae to O_3_-transformed versus original host VOC blends, which were created by mixing *P. xylostella*-induced cabbage volatile blends with O_3_ or clean air. We tested two O_3_ levels, 50 and 100 ppb, selected as typical of rural and urban areas. Cabbage plants were pre-infested for 40 h with 20 mixed instar *P. xylostella* larvae per plant. Larvae were then removed, and roots were carefully washed and inserted into water-filled bottles.

The experimental setup is shown in Fig. 1. In brief, compressed air purified through a charcoal filter, was pushed at 600 ml min^−1^ into a glass desiccator (22.4 l) containing the odour source, which comprised four plants pre-infested with *P. xylostella*. Outlet air from the source chamber was split evenly into two glass desiccators (22.4 l), with one chamber supplemented with O_3_-enriched air and the other with O_3_-free air. Both O_3_-enriched and free airflows (300 ml min^−1^ each) were generated by a calibration O_3_ generator outlet of the O_3_ analyser (Dasibi1008-RS; Dasibi Environmental Corp., Glendale, CA, USA) using charcoal-filtered air. The O_3_-free air was produced by passing the air through an O_3_ scrubber [potassium iodide (KI) coated copper tube]. Outlet air from each reaction chamber (600 ml min^−1^) was passed through a KI O_3_ scrubber (which does not remove VOCs) and into an arm of the Y-tube olfactometer. After assembly, the system was left for 3 h to allow for stabilization, and then Y-tube bioassays were conducted as in experiment 1. O_3_ levels and flow rates at the outlets of reaction chambers were checked regularly. Experiments in which O_3_-free air was fed into both reaction chambers were also done to test the symmetry of the system. For each O_3_ level, experiments were repeated four times on different days, with 24–36 larvae tested per replication. During the course of behavioural assessment, VOCs exiting the reaction chambers were collected.

#### Experiment 3: Effects of O_3_-initiated reaction products on orientation responses of P. xylostella larvae

Not only can O_3_ degrade biologically active volatile compounds to form new reaction products, their oxidation products can also form low-volatility compounds and SOA particles. To assess the potential effects of oxidation products, in particular SOA particles, on host orientation of *P. xylostella* larvae, we employed Teflon membrane filters (Pall Life Science, 25 mm diameter, 1.0 μm pore size; Pall Corp., Ann Arbor, Michigan, USA), which have been widely and successfully used in the field of atmospheric chemistry to sample aerosol particles^49^, to collect O_3_-initiated SOA particles. An earlier study has shown that O_3_-initiated oxidation of cabbage VOCs leads to the formation of SOA^40^. Here, we would expect that some low and/or non-volatile oxidation productions of cabbage VOCs, particularly when bundled together to form SOA, may condense onto the Teflon filters, and consequently influence larval behavioural responses.

The chemical reaction took place in a way similar to experiment 2, except that inside the source chambers there were three cabbage plants with the pots wrapped in aluminium foil, and the flow rates into the reaction chambers from both the source chamber and the O_3_ generator were 1000 ml min^−1^. The outgoing airflow of each reaction chamber passed through a KI O_3_ scrubber and was then divided into two, with 1800 ml min^−1^ used for SOA collection and the remaining 200 ml min^−1^ for VOC collection. After a 2-h stabilization period, SOA and VOC collections began and lasted for 3 h. SOA collection was done by drawing air thought the Teflon filters fitted in stainless-steel filter holders (Pall Life Science, 25 mm diameter; Pall Corp., Ann Arbor, Michigan) with a vacuum pump. Immediately following SOA collection, Teflon filters were used as odour sources for dual-choice tests.

The filter-disc choice tests were performed in glass Petri dishes (9 cm diameter). Two differently treated Teflon filters were placed 4 cm apart in a Petri dish. A larva was released halfway between the two filters and was given 5 min to choose between them from the moment it started moving. A choice was noted when the larva walked onto either of the two filters and stayed there for at least 5 sec. Otherwise, the larva was classified as non-responsive. We further recorded the time larvae spent walking on the filters. Each larva was tested only once using a clean Petri dish, and the position of the two filters was switched after each trial to compensate for potential side preferences.

Three comparisons were made with cabbage VOCs: filters exposed to *P. xylostella*-induced VOCs (iVOC) mixed with 50 ppb O_3_ versus clean air, filters exposed to iVOC mixed with 100 ppb O_3_ versus clean air, and filters exposed to constitutive VOCs (cVOC) mixed with 100 ppb O_3_ versus clean air. In addition, a comparison was made using filters exposed to iVOCs of broccoli plants mixed with 100 ppb O_3_ versus clean air. Each choice test was replicated 10 times with new odour sources and new filters, and 10–12 larvae were tested per replicate. To determine if volatiles can condense onto the surface of the filters and then affect olfactory responses of larvae, we compared the preference of larvae for filters exposed to iVOC or cVOC against filters exposed to clean air. It should be noted that since each pair of filters was repeatedly tested with 10–12 larvae, it is possible that the chemical traces left by the walking of the previous test larvae may influence the decision of the later tested larvae. However, our finding that larvae preferred iVOC-exposed to clean air-exposed filters, but spent similar amounts of time on them indicates that the chemical cues left by larvae play little if any role in the observed behavioural responses of larvae (see results).

### Headspace collection and analysis of volatiles

For all three experiments, volatiles exiting the source chambers (experiment 1) or reaction chambers (experiments 2 and 3) were collected. Volatiles were trapped for 1 h (experiments 1 and 2) or 3 h (experiment 3) by pulling air at a rate of 200 ml min^−1^ through a stainless steel tube packed with 150 mg Tenax TA and 150 mg Carbopack B (Markes International Ltd, Llantrisant, UK).

VOCs were analysed with GC-MS (Agilent 7890A GC and 5975C VL MSD; New York, USA). Trapped compounds were desorbed with an automated thermal desorber (TD-100; Markes International Ltd, Llantrisant, UK) at 250 °C for 10 min, cryofocused at −10 °C and then transferred to an HP-5 capillary column (50 m × 0.2 mm; film thickness 0.33 μm). Helium was used as a carrier gas. Oven temperature was held at 40 °C for 1 min, then programmed at 5 °C min^−1^ to 210 °C, and then at 20 °C min^−1^ to 250 °C under a column flow of 1.2 ml min^−1^. Tentative identification was made by comparison of spectra with the mass spectral databases NIST 2005 and Wiley 8^th^ edition spectral library, and verified with authentic standards when available. Standards were loaded into the Tenax tubes, of the same type used in the sample collections, using a calibration solution loading rig (Markes International Ltd, Llantrisant, UK), and were analyzed in the same way as the samples. For quantification, peak areas of the target ions that are specific to the compound classes and are the most abundant in the mass spectra were integrated using the extracted ion current (EIC) chromatograms; the amount of each compound was then calculated based on external calibration curves generated with authentic standards. For compounds whose reference standards were not available, quantification was assessed relative to the external standard 1-chlorooctane.

### Statistical analysis

All choice assays were analysed with binomial tests to determine whether larval preferences differed significantly from a 50:50 distribution (p = q = 0.5, two-tailed, α = 0.05). For dual-choice filter disc bioassays, the time spent foraging by *P. xylostella* larvae on the membrane filters was analysed by Mann-Whitney *U* tests. VOC data were analysed with independent (experiment 1) or paired (experiments 2 and 3) *t*-tests. All these analyses were performed with SPSS 21.0 for Windows (SPSS Inc., Chicago, IL). Additionally, to visualize differences between different VOC blends, the total VOC profiles were analysed using Principal Component Analysis (PCA) (Simca-P 11.5; Umetrics, Umeå, Sweden). Data on individual compounds were expressed in percentages of the whole blend and processed by mean centering and unit variance scaling.

## Additional Information

**How to cite this article**: Li, T. *et al*. Atmospheric transformation of plant volatiles disrupts host plant finding. *Sci. Rep.* **6**, 33851; doi: 10.1038/srep33851 (2016).

## Supplementary Material

Supplementary Information
