# Supplementary material for: Atmospheric transformation of plant volatiles disrupts host plant finding
Source: Sci Rep. 2016 Sep 21;6:33851. doi: 10.1038/srep33851 (PMC5030639; doi:10.1038/srep33851)
Supplement: Supplementary Information [file srep33851-s1.pdf]

Supplementary information

Atmospheric transformation of plant volatiles disrupts host plant finding

Tao Li[*], James D. Blande & Jarmo K. Holopainen

Department of Environmental and Biological Sciences, University of Eastern Finland, Kuopio Campus, PO Box 1627, FI-70211, Kuopio, Finland

[*]tao.li@uef.fi

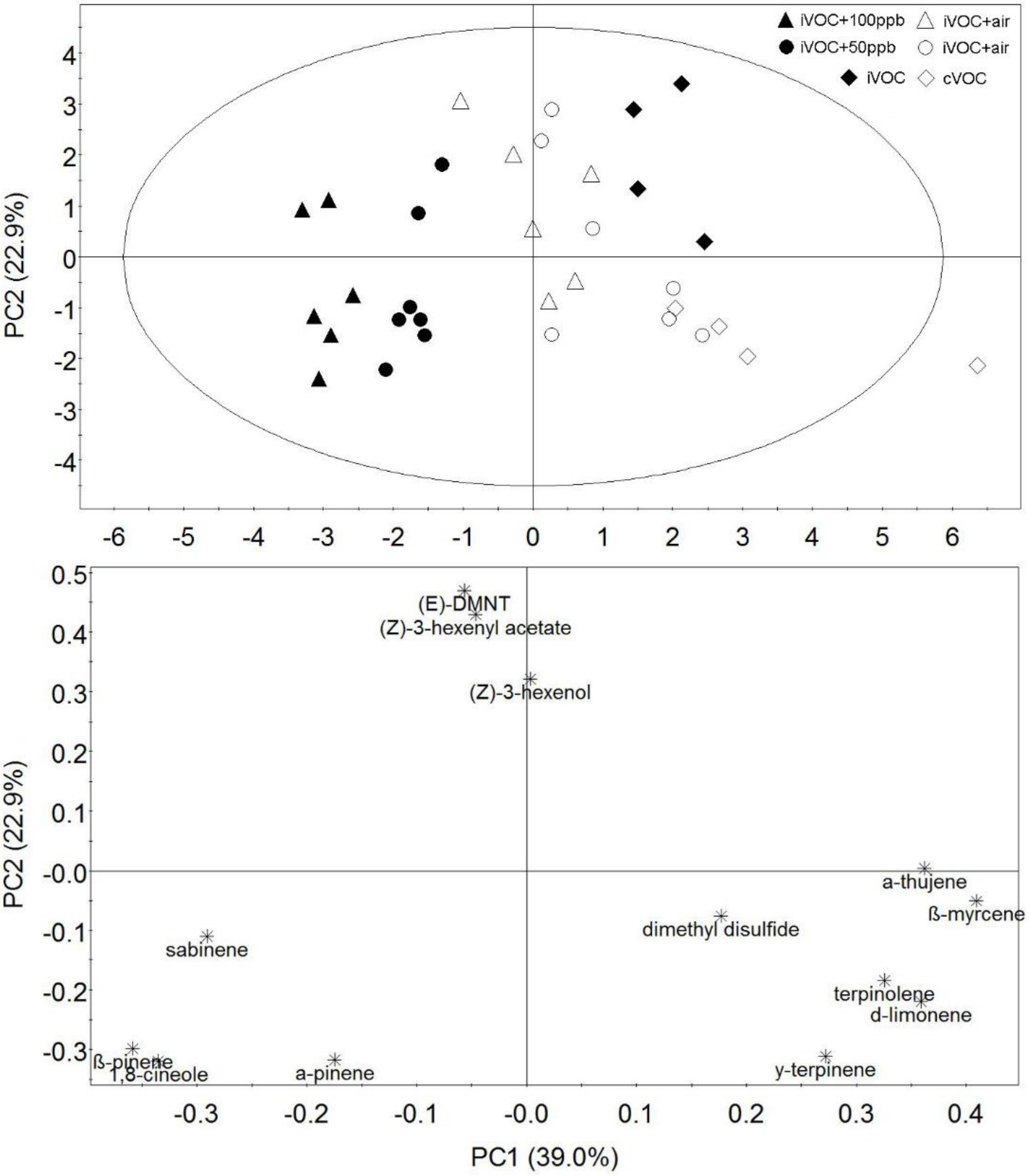

Supplementary Figure S1. Principal Component Analysis (PCA) of the VOC blends sampled in experiments 1 (diamonds) and 2 (circles and triangles). Top panel: score plot of the samples, with the percentage of explained variation displayed in parentheses. The O_3-oxidized blends, particularly when the O_3 level reaches 100 ppb, are clearly separated from the original blends. The ellipse defines the Hotelling's T^2 confidence region (95%). Bottom panel: loading plot of the two components of the PCA, showing the contribution of each of the compounds towards the model.

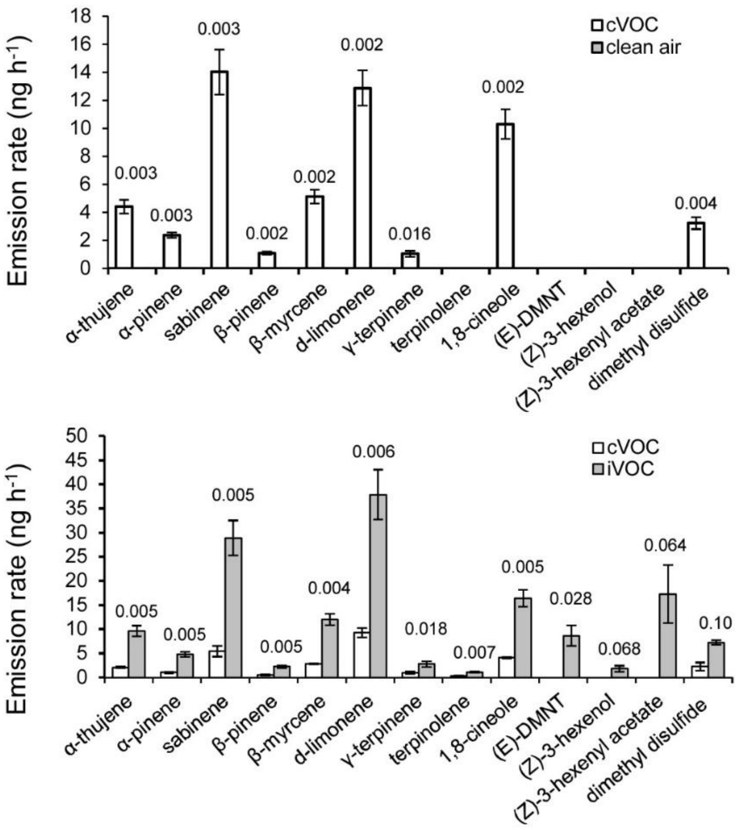

Supplementary Figure S2. VOC emission rates (mean ± se; n = 4) measured at the outlet of Y-tube olfactometers in experiment 1. *P* values by independent *t* tests are shown over the bars.

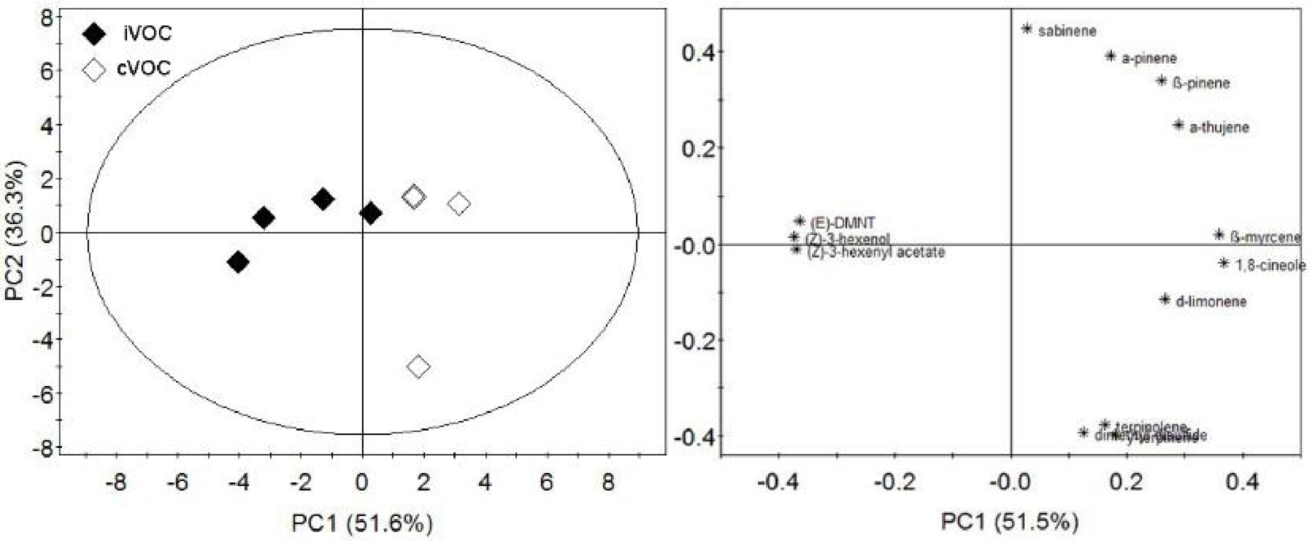

Supplementary Figure S3. Principal Component Analysis (PCA) of the VOC blends emitted by detached, control and infested plants in experiment 1. Right panel: score plot; right panel: loading plot. The percentage of explained variation is displayed in parentheses; the ellipse defines the Hotelling's T^2 confidence region (95%).

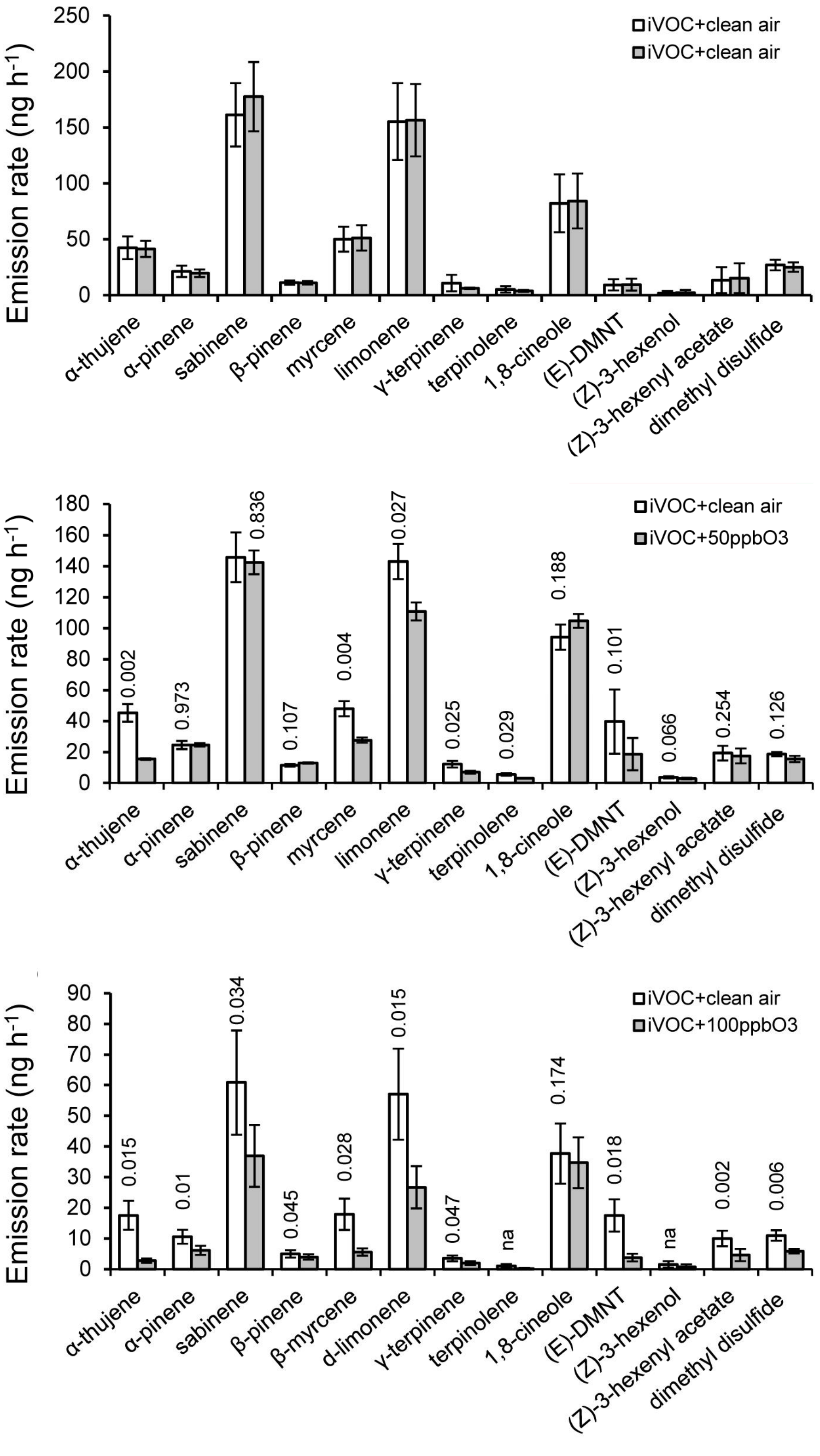

Supplementary Figure S4. VOC emission rates (mean ± se) measured at the outlet of Y-tube olfactometers in experiment 2. *P* values by paired *t* tests are shown over the bars. n = 3, 7 and 6 for top, middle and bottom panel, respectively.

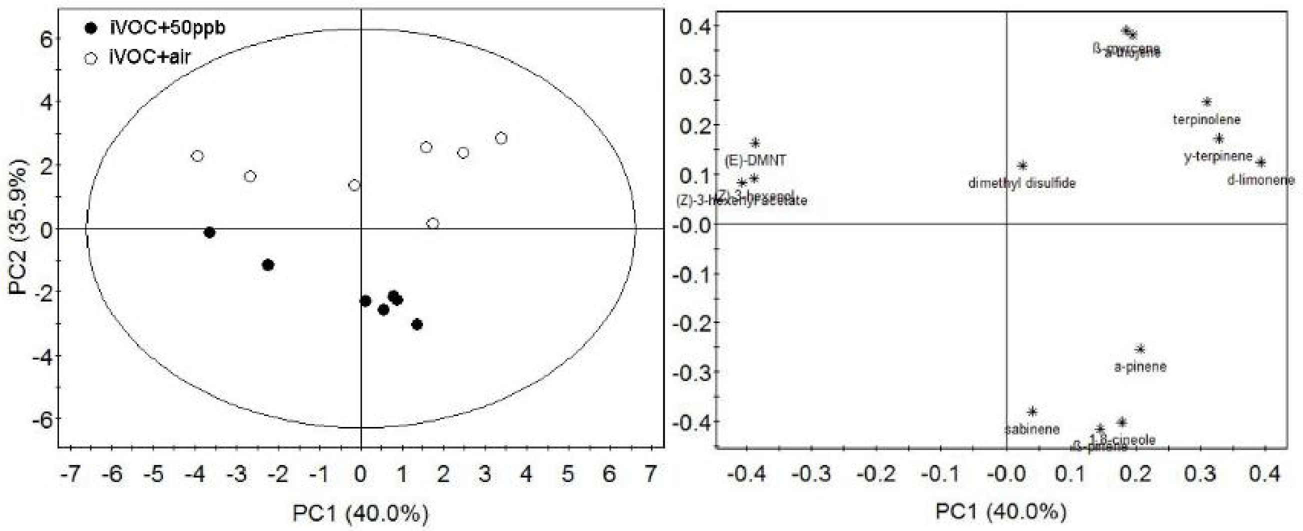

Supplementary Figure S5. Principal Component Analysis (PCA) of the iVOC blends mixed with 50 ppb O_3 and clean air in experiment 2. Right panel: score plot; right panel: loading plot. The percentage of explained variation is displayed in parentheses; the ellipse defines the Hotelling's T^2 confidence region (95%).

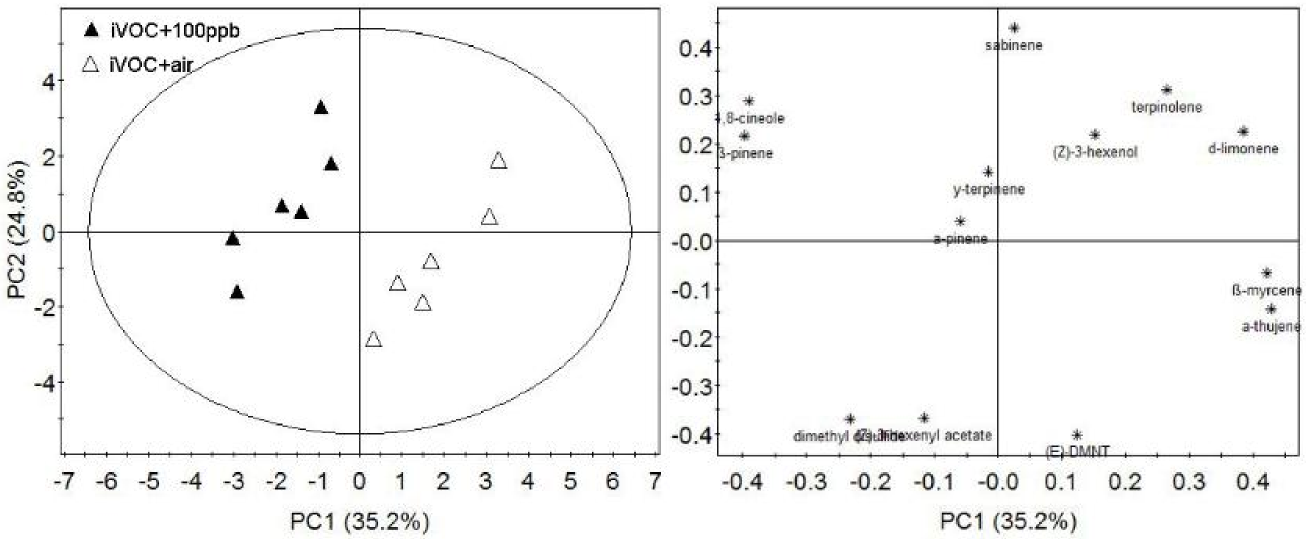

Supplementary Figure S6. Principal Component Analysis (PCA) of the iVOC blends mixed with 100 ppb O_3 and clean air in experiment 2. Right panel: score plot; right panel: loading plot. The percentage of explained variation is displayed in parentheses; the ellipse defines the Hotelling's T^2 confidence region (95%).

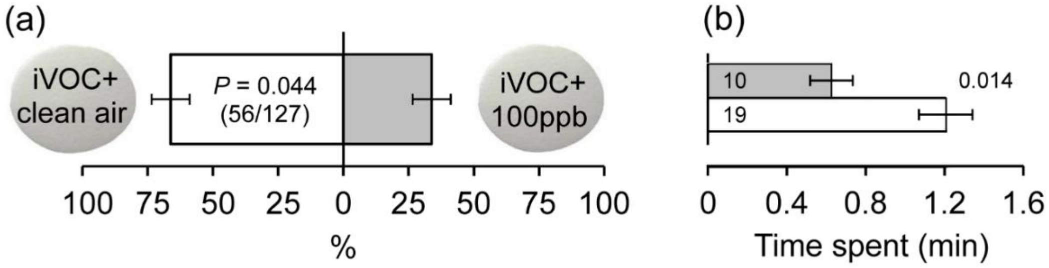

Supplementary Figure S7. Orientation preference towards (a) and time (b) spent on filters exposed to *P. xylostella*-induced broccoli VOCs mixed with 100 ppb O_3 versus clean air. Bars represent treatment mean ± se; *P* values for binomial test (a) or Mann-Whitney *U* test (b) are given.

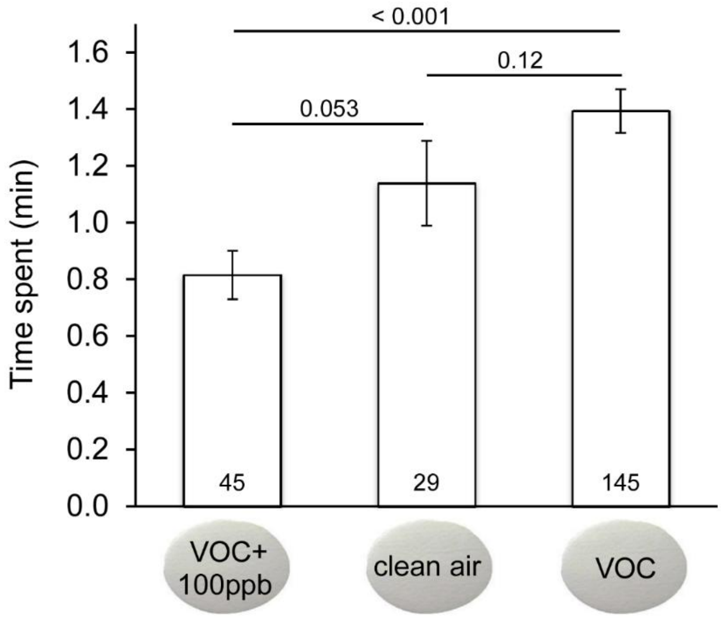

Supplementary Figure S8. Mean time (± se) spent by *P. xylostella* larvae searching on Teflon filters exposed to clean air, original VOC blends (VOC) or oxidized VOC blends by 100ppb O_3 (VOC+100ppb). Data from different filter bioassays were pooled, categorized into three treatment groups, namely, filters exposed to O_3-transformed VOCs, original VOCs, and analyzed with Kruskal-Wallis test, followed by pairwise comparisons with Mann-Whitney *U* test.
